# Behavioral Effect of Terahertz Waves in C57BL/6 Mice

**DOI:** 10.3390/bios12020079

**Published:** 2022-01-28

**Authors:** Miao Qi, Rong Liu, Bing Li, Shuai Wang, Runze Fan, Xinyi Zhao, Dehui Xu

**Affiliations:** 1 State Key Laboratory of Electrical Insulation and Power Equipment, Centre for Plasma Biomedicine, Xi’an Jiaotong University, Xi’an 710049, China; qimiao@stu.xjtu.edu.cn (M.Q.); liurong0812@stu.xjtu.edu.cn (R.L.); lb905533242@stu.xjtu.edu.cn (B.L.); shuaiwang3059@stu.xjtu.edu.cn (S.W.); fanrunze@stu.xjtu.edu.cn (R.F.); zhaoxinyi@stu.xjtu.edu.cn (X.Z.); 2 The School of Life Science and Technology, Xi’an Jiaotong University, Xi’an 710049, China

**Keywords:** terahertz, mice, behavior

## Abstract

Terahertz is a new radiation source with many unique advantages. In recent years, its application has rapidly expanded to various fields, but there are few studies on the individual effects of terahertz. In this study, we investigated the behavioral effects of terahertz radiation on C57BL/6 mice, and we conducted an open field test, an elevated plus maze test, a light–dark box test, a three-chamber social test, and a forced swim test to explore the effects of terahertz radiation on mice from a behavioral perspective. The results show that terahertz wave may increase anti-anxiety, anti-depression, and social interaction in mice.

## 1. Introduction

Terahertz (THz) wave is an electromagnetic wave with a frequency of 0.1–10 THz; the electromagnetic wave radiation wavelength range is 0.03–3 mm. THz waves are located in a unique band in the entire electromagnetic spectrum [1,2]. In recent years, with the continuous development of terahertz technology, it has been gradually studied and applied in various fields: remote sensing radar [3], material structure detection [4], data transmission [5], communication [6], security [7], and counter-terrorism [8]. In biomedicine, THz waves have the characteristics of low energy and non-ionizing radiation, and it will not cause harmful damage to biological tissues or organisms [9,10]; therefore, THz are used to identify and distinguish various biological small molecules [11], macromolecules [12,13,14], biological information extraction [15], and drug component detection [16].

The aging population is increasing, and mood disorders and neurodegenerative diseases are constantly becoming a burden on mankind. Therefore, studying the effects of terahertz irradiation on animal behavior or neurological aspects is valuable and significant for the prevention or treatment of Alzheimer’s disease, Parkinson’s disease [17,18], and depression. Olshevskaya’s team demonstrated, for the first time, that certain detection wavelengths have a highly specific effect on the structural and functional characteristics of nerve cells [19]. Kirichuk et al. used hypokinesia as a stress model for albino rats and used terahertz waves in the NO frequency range to irradiate and observe, at different times, the changes in their behavioral responses. The results showed that after 15 min of continuous irradiation, some behavioral responses were partially restored, and the effect of 30 min of irradiation was more obvious [20]. Many studies have shown that the nervous system is a sensitive target of electromagnetic radiation [21]. However, there is still no system for related research on terahertz and nerves. Terahertz has mostly been studied on neurons in vitro, but there are relatively few studies on the interaction with individual biological nervous systems. The research on the biological effects and mechanism of THz irradiation has become a new topic in the field of biology.

Animal models have been widely used in basic research in recent years, and the mouse has been used in many medical fields as a good choice in genetic research [22]; especially, the use of mice in neuropsychiatric research has rapidly grown. Mice have many basic physiological and behavioral responses that are evolutionarily conserved between species; through inference, we can study these responses to clarify behavior and support their neuromodulation as a means of using mice to understand human behavior and disease [23]. At present, several mouse disease models, such as depression [24,25], Alzheimer’s disease [26], autism [27], and schizophrenia [28], have been established. The most popular ethologically based tests include the open field, elevated plus maze, light–dark box, three-chambered social [29], tail suspension [30], and forced swimming [31]. Therefore, we can determine the effects of terahertz irradiation on the behavior of mice through experimental data on mice behavior.

This study used a terahertz source with a frequency of 0.14 THz to irradiate the head of female C57BL/6 mice and to conduct behavioral tests after a certain period of irradiation. Behavioral experiments include the open field test, elevated plus maze, light–dark box test, three-chambered social test, and forced swim test. The effects of terahertz irradiation on the behavior of C57BL/6 mice were determined by processing the experimental data. Terahertz wave may increase anti-anxiety, anti-depression, and social interaction in mice. It is of great practical significance to study the effects of terahertz on the behavior of C57BL/6 mice.

## 2. Materials and Methods

### 2.1. Animals

Eight-week-old female C57BL/6 mice were sourced from the Medical Animal Center of Xi’an Jiaotong University (*n* = 10), and they were housed under broken barrier-specific pathogen-free conditions at the Medical Animal Center of Xi’an Jiaotong University. Same sex litter mates were housed together in individually ventilated cages with four mice per cage. The control group and the treatment group were placed separately with three cages in each group. All mice were maintained on a regular diurnal lighting cycle (12:12 light:dark) with ad libitum access to food (XIETONG SHENGWU, 60Co Irradiation Sterilized Experimental Mouse/Rat Diet) and water. Chopped corn cob was used as bedding. Environmental enrichment included nesting material (XIETONG SHENGWU, Nanjing, China), PVC pipe, and shelter (Tecniplast, Varese, Italy). In this experiment, the animal behavior measurement of C57BL/6 mice used RWD’s small animal behavior analysis and recording system 3.0. The main components of the system are an experimental environment (open field, elevated plus, etc.) and video capture (camera) as well as video converters and record analysis systems. We placed a camera above or on the side of the experimental device to detect mice and camera is connected to computer software. The analysis system can remove the background and capture the location of the mice, then record the movement track, movement state, and carry out statistical analysis with the software.

### 2.2. Terahertz Generator

**Figure 1:**
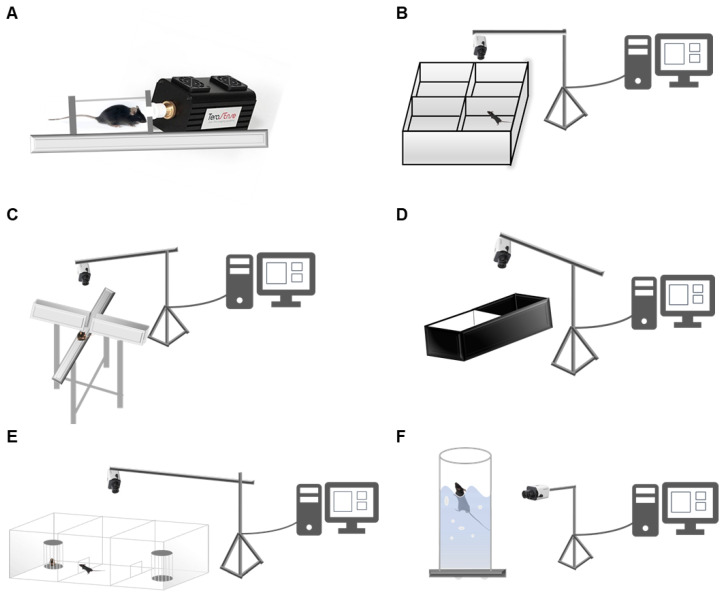
Schematics of mice behavior detection: (**A**) pattern diagram of THz-treated C57BL/6 mice, (**B**) device for the open field test, (**C**) device for the elevated plus maze, (**D**) device for the light–dark box test, (**E**) device for the three-chambered social test, and (**F**) device for the forced swim test.

The terahertz source used in this paper was a commercial terahertz generator produced in Russia. As shown in Figure 1A, the parameters of the terahertz source were working voltage of 24 V; working current of 0.3 A; overall power of 8 W; terahertz output power of 90 mW; and terahertz frequency of 0.14 THz. The heads of mice were irradiated with terahertz for 20 min for two weeks (the control group was similar to the terahertz-treated group of mice, but the terahertz source did not work) and then the behavioral tests were carried out.

### 2.3. Open Field Test

Open field tests are one of the most common models of animal behavior experiments. The main principle is to observe the duration and frequency of some behaviors and movement states of the experimental object, so as to reflect their independent behavior or exploration activities [32]. The number of central activities of the animals reflects the degree of anxiety; that is, the more central activities there are, the less the anxiety. The anxiety data are mainly the time and distance of the central and marginal activities. The whole open field of mice consisted of four 50 × 50 cm small squares, and the height of the boxes was 30 cm. The camera was located directly above the box, collecting and recording information on the movement of the mice and uploading the data to the processing system. At the beginning of the experiment, the mice were placed in the center of the bottom of the grid, the movement of the mice was observed, and the camera and timing were taken at the same time. The test stopped after 5 min of observation, the mice were taken out, and the open field reaction tank was cleaned to avoid other information, such as the excreta of the experimental animals, affecting the next test results. The open field experimental device is shown in Figure 1B.

### 2.4. Elevated plus Maze

The maze consisted of two closed arms and two open arms. The experimental mechanism is that rodents naturally love the dark, which makes them more willing to move in the closed arms when they are in the cross maze. On the other hand, mice are also curious and inquisitive, which will drive them to open their arms for activities [33]. In this device, mice will have the impulse to explore the novel environment, but at the same time, they will have the psychology of fear, which is a conflict behavior, resulting in anxiety in the mice. At the beginning of the experiment, the mice were placed in the center of the elevated cross, and the parameters, such as the number of times, resting time, and movement distance of the mice entering the open arm and the closed arm within 4 min, were counted. The more times they entered the open arm, the longer the resting time, indicating that the anxiety was lower. The elevated cross labyrinth device is shown in Figure 1C.

### 2.5. Light–Dark Box Test

The device was set-up as half a bright room and half a dark room, with a small hole in the middle, based on the characteristics of animal darkness avoidance and exploration [34]. After the experimental animal mice were put into the black and white box, they mainly moved in the black box and less in the white box, but the animal’s exploratory nature prompted it to try to move in the white box, so there would be conflicting behaviors. We put the experimental animals into the small door in the middle of the light–dark box and observed their resting time, movement distance, and entry times into the black box and white box within four min. The experimental mice showed less anxiety when they spent more time in the white box. The top view of the light–dark box experimental device is shown in Figure 1D.

### 2.6. Three-Chambered Social Test

The experimental device consisted of three areas: the left experimental area, the middle area, and the right area [35]. The principle of a three-chamber interaction experiment is that after the mice are familiar with the environment, a metal cage is placed in the experimental box; there is then a strange mouse in the cage; the time when the mouse’s head is close to the metal cage to judge the sociability of the mice is then recorded and analyzed. Generally, the sociability of experimental mice are analyzed by comparing the time when they are closest to different metal cages—one metal cage has familiar mice, and the other metal cage has unfamiliar mice. The monitoring indicators are movement distance, resting time in different areas, and times of entering the area. The three-chamber social interaction experimental device is shown in Figure 1E.

### 2.7. Forced Swim Test

The forced swim test was originally developed for rats and then modified for mice by Porsolt et al. [36,37]. In the experiment, the cylindrical water tank required for the forced swim test of the mouse was made of transparent plexiglass. The size of the water tank should be selected to ensure that the mouse cannot touch the bottom of the water tank with its feet or tail. During the swim test, the water tank should be filled with room temperature water (i.e., 23–25 °C). In the experiment, the usual test duration for mice is 6 min, but normally only the last 4 min of the test are analyzed. This is because most mice are very active at the beginning of an experiment. The experimental device is shown in Figure 1F.

## 3. Results

### 3.1. Analysis of the Open Field Test

**Figure 2:**
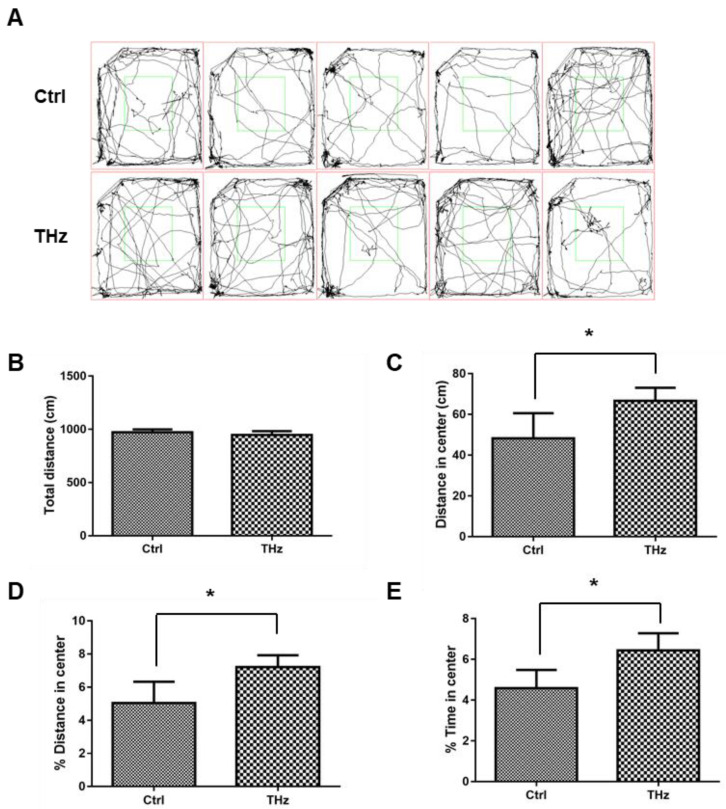
Analysis of the open field test: (**A**) moved pathways of mice in the open field test after THz treatment; changes in (**B**) total movement distance, (**C**) distance in center, (**D**) distance in center ratio, and (**E**) time in center ratio before and after THz treatment. * *p* < 0.05.

The open field test is mainly a method to analyze the autonomous behavior and anxiety of mice [38]. The main monitoring indicators include movement distance, movement distance in the center, and resting time. In this experiment, the moved pathways before and after terahertz irradiation are shown in Figure 2A,B. after terahertz treatment, the movement of mice in the control group seems no different. The movement distance of the control group in the center was 48.23 ± 12.39 cm and of the terahertz group was 66.71 ± 6.41 cm, the experimental group significantly increased (Figure 2C). The proportion of movement distance in the center and proportion of resting time in the center of the terahertz group was 1.4 times more than the control group (Figure 2D,E). In summary, when the total moving distance was close, the moving distance and the proportion of moving distance in the center of the open field in the terahertz treatment group were significantly different from those in the control group; the same is true for the resting time in the center. These results show terahertz treatment may increase anti-anxiety in mice.

### 3.2. Analysis of the Elevated plus Maze Test

**Figure 3:**
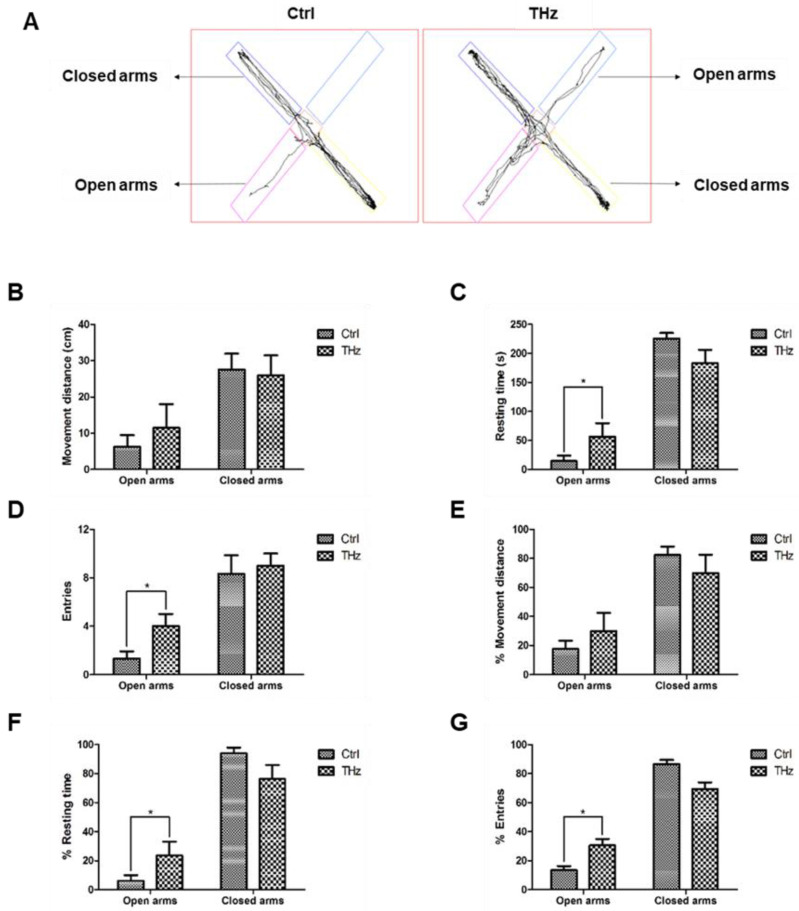
Analysis of the elevated plus maze test: (**A**) moved pathways of mice plus maze test; changes in (**B**) movement distance, (**C**) resting time, (**D**) entries, (**E**) movement distance ratio, (**F**) resting time ratio, and (**G**) entries ratio after THz treatment. * *p* < 0.05.

The elevated plus maze is one of the most widely used maze experimental model that also measures anxiety-like behavior [39]. It consists of two open arms and two closed arms crossed vertically. In this experiment, the moved pathways of the control group and the treatment group are shown in Figure 3A, and the results shown in Figure 3B–G are the movement distance, resting time, entry time, and corresponding proportion. The movement distance of the open arm and the closed arm in the control group and the experimental group were similar. The movement distance in the closed arm was much longer than that in the open arm. However, there were significant differences between the two groups in the resting time and proportion of open arm. The resting time of the control group in the open arm was 14.47 ± 9.63 s, while that of the terahertz treatment group was 56.65 ± 22.95 s, which was four times that of the control group. At the same time, the control group rarely entered the open arm, and the number of entries was about twice, while the experimental group entered the open arm about five times, which was significantly more than the control group, and the proportion of entering the open arm was also significantly greater than that of the control group. These results demonstrated that terahertz treatment may increase the anti-anxiety effect.

### 3.3. Analysis of the Light–Dark Box Test

**Figure 4:**
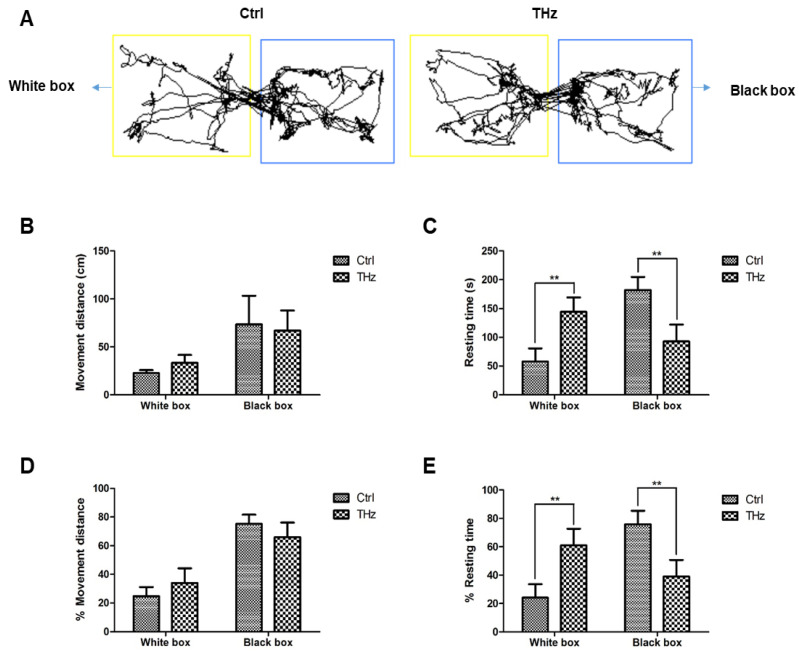
Analysis of the light–dark box test: (**A**) moved pathways of mice in the light–dark box test. After THz treatment, the mice’s (**B**) movement distance, (**C**) resting time, (**D**) % movement distance, and (**E**) % resting time were changed. * *p* < 0.05, ** *p* < 0.01.

An animal behavior model was designed based on the characteristics of rodents’ tendency towards dark and avoiding light to test anxiety. This experiment counted the movement distance of the control group and the treatment group in the black box and white box, the proportion of the movement distance in the total movement distance, and the resting time and proportion in the black box and the white box. In this experiment, its moved pathways are shown in Figure 4A, and the results shown in Figure 4B–E are the moving distance, resting time, proportion of moving distance, and proportion of resting time. The movement distance of the control group and the experimental group in the black and white box was the same, which was more in the black box than in the white box. The movement distance of the terahertz treatment group in the white box was 33.27 ± 8.29 cm and that of the control group was 22.69 ± 3.12 cm, which was more than that of the control group, but there was no significant difference between the two groups. In terms of resting time, when the total resting time was the same, the resting time in the white box was 57.86 ± 22.82 s and in the black box was 182.14 ± 22.81 s. The resting time in the black box was much longer than that in the white box. The resting time of the terahertz-treated group in the white box was 144.23 ± 25.10 s, and in the black box it was 92.80 ± 29.30 s. Therefore, after terahertz irradiation, the resting time of mice in the black and white box changed significantly, the resting time in the white box increased greatly, and the resting time in the black box significantly decreased. Results of the light–dark box test indicate that terahertz treatment may decrease anxiety in mice.

### 3.4. Analysis of the Three-Chambered Social Test

**Figure 5:**
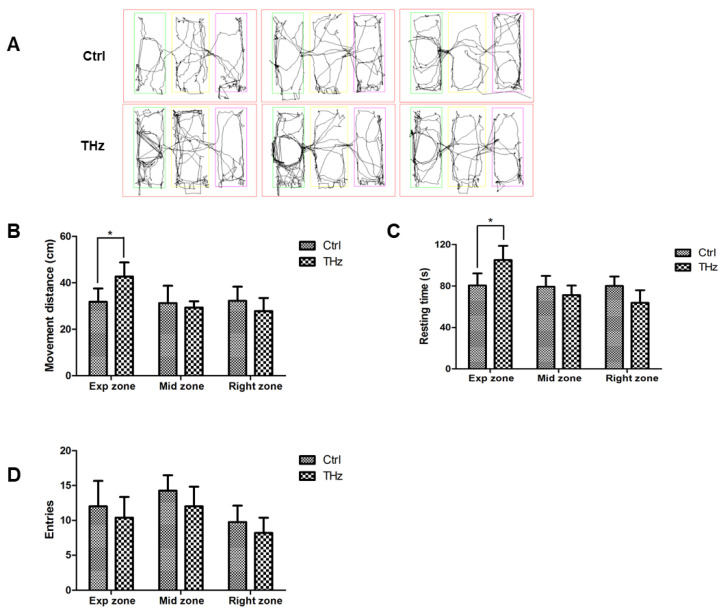
Analysis of the three-chambered social test: (**A**) moved pathways of freely moving mice in the three-chambered experimental area had a trapped mouse; after THz treatment, mice‘s (**B**) movement distance, (**C**) resting time, and entries (**D**) in three-chambered device. * *p* < 0.05.

For the three-chambered social test, a behavioral model for judging sociability and autism in mice, the main statistical parameters are the movement distance, resting time, and the proportion of entry times of mice in each area (experimental area, middle area, and right area). In this experiment, the movement pathways of experimental mice are shown in Figure 5A. It can be seen that the movement of the control group in the three regions was relatively uniform, while the terahertz treatment group had more movement pathways in the left experimental region. The overall movement shown in Figure 5B–D are the movement distance, resting time, and proportion of entry times. It can be seen that the movement distance of the control group in the three areas was very uniform, approximately 32 cm, and 31.83 ± 5.76 cm in the experimental area, while the movement distance of the THz group in the experimental area was 42.71 ± 6.07 cm, which was significantly more than that of the control group. The total experimental time of three-chamber social interaction was 240 s. There was no significant difference in the activity time of the control group in the three areas. The resting time of the experimental group in the experimental area was 105 ± 13.72 s, which was significantly longer than 80.59 ± 11.51 s in the control group, but there was no significant difference in the middle and right areas. The experimental group and the control group entered the three areas at a similar number of times, about 11 times in the experimental area, 13 times in the middle area, and 8 times in the right area. These results reveal that terahertz treatment may increase the social interaction of mice.

### 3.5. Analysis of the Forced Swim Test

**Figure 6:**
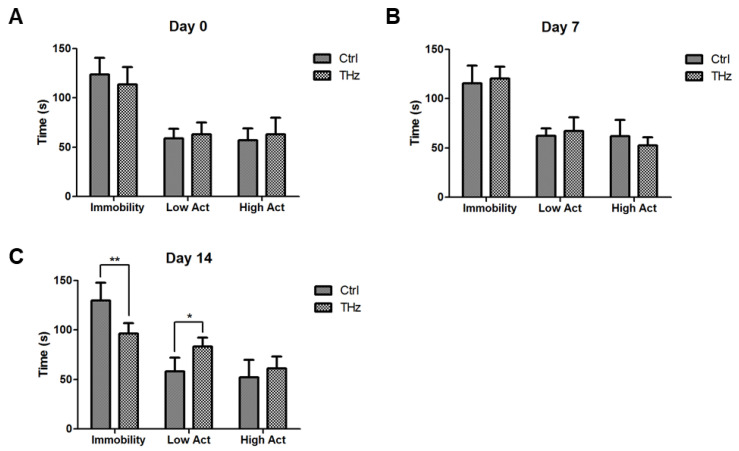
Analysis of the forced swim test. After THz treatment, mice immobility, low activity, and high activity in water changes at (**A**) day 0; (**B**) day 7; and (**C**) day 14. * *p* < 0.05, ** *p* < 0.01.

The forced swim test is one of the classical methods to detect depression. The main statistical parameters of the forced swimming experiment are the duration of the immobility, the low activity, and high activity state of the mice during the entire experimental process. In this experiment, the activity status of the mice before the beginning of the experiment (Figure 6A), on the seventh day (Figure 6B), and on the fourteenth day (Figure 6C) were detected, and the experimental duration was 4 min. From the perspective of time, at day 0 and day 7, there was no significant difference among the three states between the control group and the THz group, but at day 14, the immobility of the two groups was 129.64 ± 18.21 and 96.48 ± 10.42 s. Compared with the control group, the immobility of the THz group decreased significantly, and the duration of low activity increased. However, there was no significant difference in the state of high activity. Individually, for the control group, there was no significant difference in the duration of the three states from day 0 to day 7 to day 14. For the THz group, there was no significant difference in the duration of the three states from day 0 to day 7, but at day 14, the time of immobility decreased significantly, the time of low activity increased, and the time of high activity did not change. These results indicate forced swim test certificated terahertz treatment may increase anti-depression in mice.

## 4. Discussion

Terahertz radiation has a variety of effects depending on frequency, and experimental animals show different behavioral changes [40,41]. In this study, we adjusted the terahertz treatment time; each mouse was treated for 5 min and 10 min for 10 consecutive days, and we performed the open field test to detect anxiety (Supplementary Figure S1A,B) and the forced swim test to detect depression (Supplementary Figure S1C,D)). We found that the difference between the treatment group and the control group was not obvious, so we increased the treatment time to 20 min and found that the difference between the two groups was significantly changed. Terahertz treatment may increase anti-anxiety in mice; the open field test is routinely used to study anxiety-related behavior in mice that prefer to stay close to walls and move more peripherally, which is evident in mice that show signs of anxiety-like behavior. Mice with lower anxiety tended to spend more time in the central open area of the box [32]. After terahertz treatment, there was no significant change in total distance of the control group, but the distance in the center and resting time in the center of the terahertz group increased significantly. Campos et al. showed [42] that the elevated cross maze, perhaps the most commonly used animal model of anxiety in current practice, is raised above the ground and consists of two closed arms and two open arms. The test is based on the natural tendency of rodents to explore new environments and their natural avoidance of unprotected, bright, and high places. Use of anti-anxiety medications, such as benzodiazepines, increases open-arm exploration; moreover, after terahertz treatment, the entry times and resting time of the open arm are also increased; the resting time of the terahertz treatment group in the open arm was four times that of the control group; at the same time, the number of entries was about twice, while the experimental group entered the open arm about five times, which was significantly more than the control group. The light–dark exploration test, based on the innate aversion of rodents to places with bright light, is a model that generates an inherent conflict between their exploratory drive and their avoidance of the lit compartment; use of anti-anxiety drugs will increase the time in the light area and the number of transitions between the two areas. In this study, the resting time of the control group in the white box was 57.86 ± 22.82 s, and was 144.23 ± 25.10 s for the terahertz-treated group in the white box, which was 2.5 times that of the control group.

Moreover, terahertz treatment may increase anti-depression in mice. The forced swim test is the most commonly used assay for the study of depressive-like behavior in rodents; when an animal is placed in a container filled with water, it will first try to escape, but eventually show immobility and float without any movement. The animal is exposed to pressure, which has been proven to play a role in the trend of major depression. Drug treatment with antidepressants before the test has been proven to reduce immobility in the forced swim test. Therefore, it can be used as a rodent model to predict the clinical efficacy of antidepressants [43]. For the THz group, there was no significant difference in the duration of the three states from day 0 to day 7, but at day 14, the time of immobility decreased significantly, the time of low activity increased, and the time of high activity did not change; therefore, terahertz prevents depression. 

Furthermore, terahertz treatment may increase social interaction of mice. Sociability was defined as the test mice spending more time in the room containing the new mouse than in the room containing the inanimate new object [44]. Social interaction testing involves placing familiar or unfamiliar male or female rodents in a new environment and monitoring their exploration and social behavior, which contains the three-chambered social approach test [35]. This test is judged by monitoring the movement distance, resting time, and entry times of mice in the three areas. The experimental results showed that the movement distance and resting time of the terahertz treatment group in the experimental area were significantly longer than in the control group. But there was no significant difference in the number of entries across the three zones for each group.

In addition, terahertz treatment may not cause aggression in mice. Aggressive bite is the most prominent and significant element in mice aggression [45]. Bites were divided into three categories: (1) direct bites; (2) focused bites to the wire cage containing the unfamiliar conspecific; and (3) unfocused bites to the rest of the apparatus [46]. In the experiment, four mice were in a cage, and within 2 weeks of terahertz treatment, the mice did not suffer from bites, such as to the tail, the snout, the belly, or biting the cage. 

## 5. Conclusions

We mainly explored the behavioral changes of C57BL/6 mice treated with terahertz. The heads of C57BL/6 mice were irradiated with a terahertz source with a frequency of 0.14 THz for 20 min each time, once a day for two weeks, and then animal behavior experiments were carried out after terahertz irradiation. The open field test, elevated plus maze, light–dark box test, three-chambered social test, and forced swim test were carried out. The results show that terahertz wave may increase anti-anxiety, anti-depression, and social interaction in mice.

## Data Availability

Not applicable.

## Supplementary Materials

The following are available online at https://www.mdpi.com/article/10.3390/bios12020079/s1, Figure S1: Analysis of the open fielded test and forced swim test after THz treatment. (A) moved pathways (B) and distance in center in the open field test; (C) immobility time (D) and swimming time in forced swim test.
