# Supplementary material for: Behavioral Effect of Terahertz Waves in C57BL/6 Mice
Source: Biosensors (Basel). 2022 Jan 28;12(2):79. doi: 10.3390/bios12020079 (PMC8869163; doi:10.3390/bios12020079)
Supplement: Supplementary file 1 [file biosensors-12-00079-s001.zip › biosensors-1565623-supplementary.pdf]

biosensors

Supplementary

Behavioral Effect of Terahertz Waves in C57BL/6 Mice

Miao Qi [1, 2], Rong Liu [1,2], Bing Li [1,2], Shuai Wang [1,2], Runze Fan [1], Xinyi Zhao [1,2] and Dehui Xu[1]*

[1] State Key Laboratory of Electrical Insulation and Power Equipment, Centre for Plasma Biomedicine, Xi'an Jiaotong University, Xi'an, Shaanxi, 710049, China; qimiao@stu.xjtu.edu.cn (M.Q.); liurong0812@stu.xjtu.edu.cn (R.L.); lb905533242@stu.xjtu.edu.cn (B.L.); shuaiwang3059@stu.xjtu.edu.cn(S.W.); fanrunze@stu.xjtu.edu.cn (R.F.); zhaoxinyi@stu.xjtu.edu.cn (X.Z.)

[2] The school of Life Science and Technology, Xi'an Jiaotong University, Xi'an, Shaanxi, 710049, China.

* Correspondence: dehuixu@mail.xjtu.edu.cn

Citation: Qi, M.; Liu, R.; Li, B.; Wang, S.; Fan, R.; Zhao, X.; Xu, D. Behavioral Effect of Terahertz Waves in C57BL/6 Mice. *Biosensors* **2022**, *12*, 79. https://doi.org/10.3390/bios12020079

Received: 05 January 2022
Accepted: 26 January 2022
Published: 28 January 2022

Publisher's Note: MDPI stays neutral with regard to jurisdictional claims in published maps and institutional affiliations.

Copyright: © 2022 by the authors. Submitted for possible open access publication under the terms and conditions of the Creative Commons Attribution (CC BY) license (https://creativecommons.org/licenses/by/4.0/).

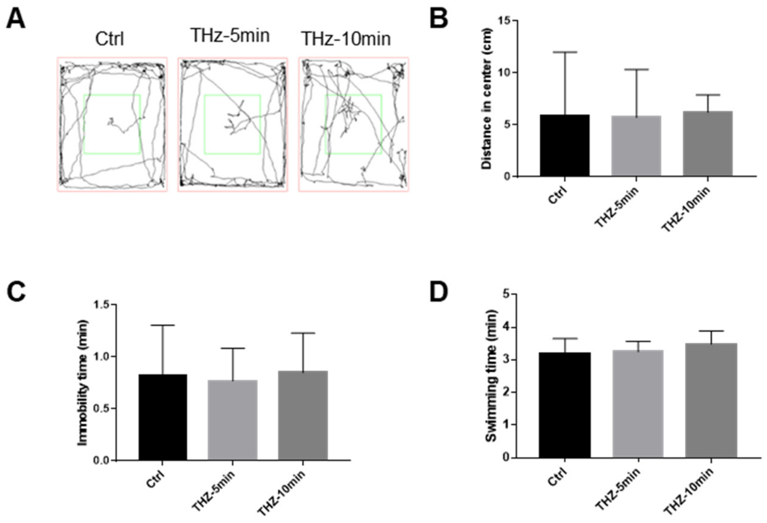

Figure S1. Analysis of the open fielded test and forced swim test after THz treatment. (**A**) moved pathways (**B**) and distance in center in the open field test; (**C**) immobility time (**D**) and swimming time in forced swim test.
